# Urolithiasis in Children—Clinical Picture, Pathogenesis, and Diagnostic Approach

**DOI:** 10.3390/biom16010119

**Published:** 2026-01-09

**Authors:** Justyna Pięta, Michał Szyszka, Patryk Lipiński, Piotr Skrzypczyk

**Affiliations:** 1 Student Scientific Group, The Department of Pediatrics and Nephrology, Medical University of Warsaw, 02-091 Warsaw, Poland; justwp@o2.pl; 2 Department of Pediatrics and Nephrology, Doctoral School, Medical University of Warsaw, 02-091 Warsaw, Poland; 3 Department of Pediatrics, Medical Center of Postgraduate Education, 01-813 Warsaw, Poland; patryk.lipinski.92@gmail.com; 4 Department of Pediatrics and Nephrology, Medical University of Warsaw, 02-091 Warsaw, Poland; pskrzypczyk@wum.edu.pl

**Keywords:** urolithiasis, children, metabolic disorders, tubulopathies, urinary tract infections, urinary tract malformations, diagnostic

## Abstract

As in adults, urolithiasis is a significant health problem in children from an early age, having a very negative impact on health and quality of life and potentially leading to kidney function impairment. The occurrence of deposits in the urinary tract in a child is almost always the result of significant predisposing factors, including metabolic defects involving the kidney or the entire body (often inherited in a Mendelian fashion), urinary tract defects, or urinary tract infections. Among metabolic disturbances, idiopathic hypercalciuria, preceded by hypocitraturia, is the most common one. Any child with nephrolithiasis requires a careful metabolic evaluation, including blood tests, urinalysis, and, in many cases, molecular diagnosis. This narrative review presents the epidemiology, pathophysiology, and diagnostic process in children with nephrolithiasis. Special emphasis is put on pathophysiological pathways leading to metabolic kidney stone disease and metabolic diagnostic steps in children with urolithiasis, as metabolic disturbances are the most common cause of recurrent urolithiasis in Europe and North America. Nephrolithiasis should be treated as a symptom of renal or systemic disorders, and in every child, the cause of these disorders should be sought to prevent recurrence.

## 1. Introduction

Urolithiasis is widely regarded as a disease that manifests exclusively in adults; nevertheless, 1 in 10 patients with kidney stones is a child [1]. Of note, urolithiasis accounts for 10–12% of cases of acute kidney injury (AKI) caused by obstruction, and represents only about 1–2% of all AKI events [2]. Though in children, some data suggest that obstructive stones may account for as much as 30% of all cases of pediatric AKI [3]. Finally, the risk of chronic kidney disease (CKD) and end-stage kidney disease (ESKD) is substantially increased in patients with monogenic stone diseases such as primary hyperoxaluria, cystinuria, Dent disease, and adenine phosphoribosyltransferase deficiency [4]. The negative impact of urolithiasis on quality of life, especially in the case of recurrent renal colic, should not be overlooked. The pain caused by a stone passing through the urinary tract is considered one of the most severe types of pain experienced by humans [5].

Despite this, there remains a paucity of both pediatric guidelines and standardized protocols for the diagnosis and treatment of urolithiasis in children. The condition may involve the bladder (cystolithiasis) or the kidney (nephrolithiasis). Nephrocalcinosis is a term referring to calcium salt deposition within the renal parenchyma [6].

When discussing urolithiasis, it is essential to distinguish between two clinical states: an acute episode of renal colic, which is often the first symptom requiring immediate medical intervention, and a predisposition to stone formation, indicated by laboratory abnormalities detected during routine check-ups, such as incidental microscopic hematuria. While the latter also necessitates a thorough diagnostic evaluation, it does not require urgent medical help, unlike an acute renal colic episode [7].

Since pediatrics covers patients from birth to adulthood, symptoms, causes, and diagnostic approaches vary significantly by age. While symptoms, causes, and diagnostics in adolescents are similar to those in young adults, in younger children (especially ≤ 2 years), on the one hand, the causes (primarily genetic background) must be intensively sought, and on the other hand, diagnostics pose many challenges (limitations in the amount of blood that can be collected or difficulties in performing a 24 h urine collection) [6,8].

It should be emphasized that kidney stones in children may be the first symptom of a systemic disease whose symptoms extend far beyond the urinary system, e.g., primary isolated hyperparathyroidism or MEN1 or MEN2A syndromes (multiple endocrine neoplasia), which may be accompanied by other endocrinopathies like pheochromocytoma or inborn errors of metabolism, e.g., primary hyperoxaluria or glycogen storage disease type I.

This article explores the epidemiology, types of urolithiasis, pathogenesis, and diagnostic approaches. Due to the extensive scope of the topic, treatment strategies are not included in this discussion.

The uniqueness of our narrative review lies in its broad, modern approach to the problem. The authors of the manuscript address both pediatric nephrology and congenital metabolic disorders, thereby enabling an interdisciplinary and holistic view of metabolic disorders in pediatric urolithiasis.

## 2. Epidemiology

Children represent 2–10% of patients with urolithiasis [9]. The prevalence of urolithiasis in adults varies worldwide, ranging from 9 to 10 percent in Europe, 12 to 15 percent in the United States, 1 to 5 percent in Asia, and up to 20 percent in Arab countries [8]. The highest incidence of kidney stones in both adults and children occurs in Saudi Arabia [8]. In Europe, nephrolithiasis associated with metabolic defects is more prevalent, with a higher incidence of calcium-containing stones. In Africa and South Asia, uric acid stones are more common, and bladder stones are also more frequently observed, mainly due to dietary factors and hot climates.

There are areas in the world that are particularly affected by kidney stones, known as stone belts. The American stone belt covers the southeastern states of the US. In contrast, the Asian stone belt begins in northeastern Africa and stretches across the Arabian Peninsula and the Indian Peninsula to Indochina. It is postulated that the increased incidence of urolithiasis in these areas is due to the warm climate, dietary and genetic factors, e.g., the high prevalence of distal tubular acidosis in Thailand [8]. Pediatric epidemiological data suggest a higher incidence of urolithiasis in rural areas and in environments with lower socioeconomic status, similar to adults. A study of data from South Carolina, USA, revealed that urolithiasis was more prevalent in rural counties than in urban ones [1]. It is worth noting that bladder stones are more common in these areas than in developed countries [8].

The incidence of kidney stones varies with age; it is infrequent in young children, more often detected incidentally on ultrasound performed for other reasons, and increases with age. There is a particular increase in frequency during puberty, reaching the frequency observed in adults in the oldest teenagers, in whom environmental factors also begin to predominate in the pathogenesis [10,11].

In numerous countries, including Kuwait, Saudi Arabia, Japan, and Taiwan, there is evidence of a seasonal occurrence of urolithiasis, with a notable increase during the summer months. This phenomenon may be related to increased urine concentration and elevated vitamin D synthesis, which are induced by UV radiation on the skin [12,13].

Moreover, boys are more commonly affected, although an increasing incidence of urolithiasis in girls, particularly those aged 10–17 years, has been noticed in recent years [14]. The male-to-female ratio appears to be higher in children of White origin compared to Black Americans and Hispanics [8]. The gender distribution of pediatric patients with urolithiasis showed significant variation across age groups, with a higher prevalence in boys during the first decade of life, and a predominance of girls in the second decade [15].

A comparison of the incidence of kidney stones by race shows that among children living in the same area, Caucasians are more frequently affected than African Americans, with a ratio of up to 4 to 1. It appears that the differences are due to both genetic and environmental (dietary) factors [8].

There is a systematic rise in the incidence of urolithiasis across all regions of the world. This may be related to the enhanced availability of imaging tests, particularly ultrasound, and the detection of asymptomatic urolithiasis. In adolescents, as in adults, lifestyle changes and a higher prevalence of obesity may be responsible, and this association has been demonstrated in both populations [16,17]. However, in young children, the leading causes of urolithiasis are anatomical, metabolic, and genetic defects [18]. Another worrying factor contributing to the growth in the incidence of urolithiasis is a warming climate [19]. Episodes of urolithiasis increase following periods of high temperature, which is related to increased urine concentration [13,20]. According to a study by Sas et al. conducted in South Carolina over 12 years (1996–2007), the incidence of urolithiasis in children increased from 7.9 per 100,000 to 18.5 per 100,000. The rising incidence affected both boys and girls, across all ethnicities and age groups, but was most pronounced among adolescents [1].

## 3. Types of Urolithiasis

Urolithiasis in children can be divided according to [21]:

− Location—we refer to nephrolithiasis, i.e., the formation of stones in the upper urinary tract (usually on the renal papillae), and cystolithiasis, i.e., bladder stones. Over the past 100 years, the incidence of bladder stones has decreased in the developed world, primarily due to changes in dietary habits, including increased phosphate intake [8]. Bladder stones were found in only 2.5% of cases in a retrospective study of 300 Caucasian children between 2006 and 2017 [22].
− Etiology—we distinguish between metabolic, infectious, and urolithiasis caused by environmental factors, e.g., drug-induced. A retrospective analysis of the medical records of children aged 0–3 years revealed that urinary tract defects and infections co-occurred with urolithiasis in more than 70% of the patients followed [23]. In contrast, other studies have detected metabolic abnormalities in 90% of children with urolithiasis [13,22,24]. Furthermore, it should be emphasized that it is often a combination of the factors mentioned above that predisposes individuals to urolithiasis [8].
− Stone composition is considered to be the most crucial division. The deposits in the urinary tract consist of inorganic elements (crystals) and organic elements (matrix), which include proteins and carbohydrates [25]. Based on the chemical composition of the inorganic component, urinary tract deposits are divided into [8,26]: calcium oxalate and mixed calcium oxalate-phosphate lithiasis (70–80% in Poland and other European countries), uric acid stones (10–15%) struvite (magnesium-ammonium phosphate) stones (5–7%), carbonate apatite stones (4–6%) and other (brucite, cystine, xanthine) (1–2%).

## 4. Pathophysiology

The process of deposit formation is a complex physico-chemical-biological phenomenon. For precipitation and growth of deposits, several factors must be fulfilled [27]:

− increased urinary concentration of deposit components (promoters of crystallization, e.g., calcium, oxalate)
− decreased urinary concentration of crystallization inhibitors
− adequate urinary pH to promote crystallization

The process of stone formation can be divided into the following stages: nucleation, crystal growth, aggregation, and retention. Any damage to the uroepithelium, such as infection, the presence of a foreign body, or Randall’s plaques, can serve as an initiation point for stone formation. Randall’s plaques are composed of calcium phosphate crystals that form within the basement membrane of the thin loops of Henle. As aggregation persists, these crystals coalesce into plaques and migrate to the renal papillary interstitium. If the epithelium over the plaque is disrupted, calcium oxalate deposits are added to the process, and these accumulate to form stones. This mechanism is critical in the development of most cases of idiopathic calcium oxalate nephrolithiasis [26,28].

Most cases of urolithiasis in childhood are metabolic stones, which implies that patients have a genetically determined defect leading to increased concentrations of crystallization promoters or decreased concentrations of crystallization inhibitors [8]. The genetic predisposition is compounded by environmental factors, such as a poor diet, low fluid intake, and immobility [29].

The solubility of urine components is also significantly affected by urine pH. It depends on many factors, including diet (in general, a diet rich in milk and vegetables alkalizes the urine, whereas a diet rich in meat and eggs acidifies it). Phosphate stones (calcium phosphate and ammonium-magnesium phosphate) are deposited in alkaline urine, and stones made up of weak organic acids (uric acid and cystine) are deposited in acidic urine [27].

The most significant inhibitors of urinary tract deposit formation are citrate and magnesium [30]. In recent years, there has been a growing emphasis on the role of genetically determined differences in the concentrations of proteins that act as endogenous inhibitors of crystallization, such as fetuin-A, osteopontin, lithostathine, and bacunin [31,32,33]. Inhibiting and promoting factors of stone formation are listed in Table 1.

The phrase ‘children are not small adults’ applies more than ever to urolithiasis. Congenital genetic metabolic abnormalities are much more common in children than in adults, and their prevalence increases with decreasing age. On the other hand, environmental factors acquired in children (e.g., diet, obesity) and congenital predispositions in adults with urolithiasis should not be underestimated [34,35].

## 5. Excretory Disorders in Urolithiasis

### 5.1. Hypercalciuria

Hypercalciuria (i.e., excessive excretion of calcium in the urine) is the most common metabolic disorder associated with pediatric kidney stone disease [11]. In a study of 300 pediatric patients, 89.3% had biochemical abnormalities, of which idiopathic hypercalciuria was detected in 47.01% [22]. Calcium crystallizes in the urinary tract with oxalate or phosphate anion, which is the main component of two-thirds of all deposits (80–90% of deposits unrelated to urinary tract infections) [36]. Additional factors in patients with hypercalciuria that predispose them to deposit precipitation include low urine volume (resulting from low fluid intake or a hot climate), coexisting hypocitraturia and/or hypomagnesuria, high urine pH (which predisposes to calcium phosphate precipitation), and a sodium-rich diet. A high-sodium diet increases urinary calcium excretion due to the mechanism of lower proximal tubular reabsorption of sodium [37]. The majority of calcium (Ca^2+^) reabsorption in the proximal tubules is passive and paracellular, driven by the active transcellular transport of sodium (Na^+^). NHE3 (sodium-hydrogen exchanger isoform 3) is primarily responsible for Na^+^ reabsorption in the proximal tubules, thereby establishing an osmotic gradient that facilitates water reabsorption. This water movement either increases the luminal Ca^2+^ concentration, creating a concentration gradient that favors paracellular Ca^2+^ reabsorption [38].

The majority of serum calcium filtered by the glomerulus (over 60%) is reabsorbed in the proximal tubule via a paracellular pathway involving tight junction proteins called claudins, which help determine ion selectivity [39]. Calcium reabsorption in the proximal tubule is decreased in patients with hypercalciuria; however, both dietary and pharmacological treatments can enhance this process in this segment [40]. The movement of water and sodium primarily drives this reabsorption. An additional 30% to 35% of calcium is reabsorbed in the thick ascending limb of the loop of Henle via claudin proteins—claudin-14, claudin-16, and claudin-19 [41]. Activated vitamin D (1,25-dihydroxy vitamin D) reduces claudin activity. Claudins also facilitate magnesium transport, and their upregulation can be triggered by low magnesium levels (hypomagnesemia). The distal convoluted tubule and collecting ducts further control calcium reabsorption and excretion [39]. The distal convoluted tubule (DCT), where reabsorption is transcellular rather than paracellular, receives only a small fraction (10–15%) of the filtered calcium [42].

The most common cause of hypercalciuria is idiopathic hypercalciuria (IH)—a heterogeneous group of genetically determined defects that result in increased urinary calcium excretion with normal serum calcium levels. It is the most common abnormality found in patients with recurrent nephrolithiasis; according to some data, IH can occur in up to 3–10% of people (although not all people with IH form deposits). IH can be sporadic or familial (usually autosomal dominant).

For over 50 years, attempts have been made to understand the pathophysiology and classify patients with IH. Traditionally, IH patients have been divided into three groups: absorptive, renal, and resorptive IH. Sometimes other subtypes were also distinguished, such as “true idiopathic hypercalciuria” and IH dependent on renal phosphate loss. It has been postulated that patients with absorptive IH have excessive calcium absorption from the gastrointestinal tract. In contrast, patients with renal IH have reduced calcium excretion in the urine (“elevated renal threshold for calcium”), and in patients with resorptive IH, excess calcium in the urine results from excessive bone resorption [29,37,43]. To differentiate between types of IH, the Pak test (modified by Stapleton for use in pediatrics) was performed, which involved administering a standard oral dose of calcium and assessing calciuria before and after calcium loading [44]. Depending on the test result, therapeutic measures were implemented. Although this test is still used, it is not widely recommended. Where possible, molecular analysis of the patient is recommended.

In recent years, genetic studies have revealed that many patients with IH have pathogenic variants in genes responsible for vitamin D metabolism (CYP24A1—the gene encoding the enzyme 24-hydroxylase, which inactivates vitamin D) or tubular phosphate transport (SLC34A1 or SLC34A3—sodium-phosphate cotransporters in the proximal tubule, NaPi2a and NaPi2c, respectively) [37].

Biallelic pathogenic variants in the *CYP24A1* gene cause deficiency of vitamin D 24-hydroxylase and lead to high vitamin D concentrations (measured as serum 25OHD) despite a lack of supplementation, hypercalcemia (especially in infants), and hypercalciuria—a condition known as infantile hypercalcemia type 1 (HCINF1, #143880). At a later age, patients may present with isolated hypercalciuria, nephrocalcinosis, or recurrent kidney stones. A milder phenotype is observed in heterozygotes, although these individuals also have an increased risk of kidney stones [45,46].

In patients with pathogenic variants in *SLC34A1* or *SLC34A3*, increased urinary phosphate loss leads to inhibition of FGF23 secretion, resulting in unblocked 1-alpha-hydroxylase activity, elevated 1,25(OH)2D concentrations, increased gastrointestinal calcium absorption, positive calcium balance, and secondary hypercalciuria. Biallelic pathogenic variants in the *SLC34A1* gene are related to infantile hypercalcemia-2 (HCINF2; #616963). In HCINF2, hypercalcemia is typically observed during the neonatal and infantile periods and resolves with age [29]. Hereditary hypophosphatemic rickets with hypercalciuria (HHRH; #241530) is an autosomal recessive disorder caused by biallelic pathogenic variants in the *SLC34A3* gene. HHRH is distinct from other forms of hypophosphatemic rickets in that affected individuals present with hypercalciuria due to increased serum 1,25-dihydroxyvitamin D levels and increased intestinal calcium absorption. In general, carriers of pathogenic variants in the genes mentioned above exhibit a broad spectrum of phenotypes, with some individuals having isolated hypercalciuria.

The involvement of *CYP24A1*, *SLC34A1*, *SLC34A3*, and other genes (e.g., CASR, CLDN19, CLCN5) in the development of hypercalcemia and hypercalciuria remains unclear, given that a large proportion of patients with mild forms of the disease harbor variants of uncertain significance (VUS). The importance of such variants is unclear, but their presence in a heterozygous state has been found in families with hypercalciuria and urolithiasis [47,48].

Other causes of hypercalciuria include conditions with an excessive bone resorption (prolonged immobilization, prolonged use of corticosteroids, osteolytic metastases to bone, primary hyperparathyroidism) or excessive calcium absorption from the intestines (vitamin D3 overdose, hypersensitivity to vitamin D3—including HCINF1, and granulomatous diseases, e.g., sarcoidosis, tuberculosis, leading to excessive hydroxylation of vitamin D to the active metabolite 1,25(OH)_2_D). Hypercalciuria also occurs in many congenital tubulopathies such as Fanconi syndrome (generalized proximal tubular damage), Dent disease (#300009,#300555), Bartter syndrome (#601678, #241200, #607364, #602522, #613090, #300971), hypocalcemic hypercalciuria, familial hypomagnesemia with hypercalciuria and nephrocalcinosis (#248250, #248190), and as an effect of loop diuretics [49]. A progressive proximal renal tubulopathy with hypercalciuria, low-molecular-weight proteinuria, and nephrocalcinosis characterizes dent disease-1 (#300009). In contrast, the characteristic abnormalities of Dent disease-2 (#300555) include low-molecular-weight proteinuria and other features of Fanconi syndrome, such as glycosuria, aminoaciduria, and phosphaturia, but typically do not include proximal renal tubular acidosis. Bartter syndrome refers to a group of disorders characterized by pronounced salt wasting (due to impaired salt reabsorption in the thick ascending loop of Henle), polyuria, dehydration, hypokalemia, hypochloremic metabolic alkalosis, hyperreninemia, normal or low blood pressure, hypercalciuria, and failure to thrive. Familial hypomagnesemia with hypercalciuria and nephrocalcinosis is linked with the defect of tight-junction genes, including *CLDN16* and *CLDN19*, with the latter associated with ocular involvement. Hypercalcemia with hypercalciuria is also seen in patients with Williams syndrome [50]. The other ultra-rare cause of hypercalciuria (in severely affected patients manifested with Fanconi syndrome) is transaldolase deficiency (#606003).

Idiopathic hypercalciuria is a known risk factor for kidney stones. There is no clear correlation between the severity of calciuria and the risk of kidney stones, as this risk depends not only on calcium concentration but also on the concentration of anions: oxalates, citrates, urine pH, and the concentration of lithogenesis inhibitors, which are the main physicochemical determinants of lithogenesis [37]. In an Italian study involving 74 children with IH (mean calcium excretion: 6.1 mg/kg/24 h), 42 (57%) had microcalculi, and 4 (5%) had calculi [51]. In a Turkish study, 35 out of 131 children with IH developed nephrolithiasis during an average follow-up period of 4 years [52].

Calciuria can be assessed through a daily urine collection or, if collection is not possible (e.g., young children in diapers), in the second portion of urine collected after nighttime rest. Reliable normative values for calciuria have been established for European children [29,53] (Table 2 and Table 3).

A patient with hypercalciuria requires a comprehensive assessment of calcium and phosphate metabolism in both blood and urine (blood: creatinine, urea, sodium potassium, total calcium, phosphate, magnesium, alkaline phosphatase, 25OHD, 1,25(OH)_2_D, parathormone, acid-base balance, 24 h urine collection for calcium, sodium, magnesium, creatinine, and spot urine sample taken on the same day as blood tests for: calcium, phosphate, magnesium, and creatinine). The tests are aimed at estimating calciuria, but also at detecting other disorders that may aid in diagnosis, e.g., hypomagnesemia and increased magnesium excretion suggest FHHNC (*CLDN16* and *CLDN19* pathogenic variants), and a reduced TmP/GFR value indicates hypercalciuria dependent on renal phosphate wasting (*SLC34A1* and *SLC34A3* variants) (paragraphs 8.3, 8.4, and 8.6) (Table 4) [53,57].

### 5.2. Hyperoxaluria

There are two types of hyperoxaluria (i.e., excessive excretion of oxalate in the urine): primary (autosomally recessively inherited) inborn defects of hepatic metabolism leading to excessive endogenous oxalate production, and secondary, due to increased intestinal absorption of oxalate [6,10]. Hyperoxaluria is detected in up to 10–20% of children with urolithiasis [11,22]. Intestinal hyperoxaluria has been observed in patients with fat malabsorption and elevated intestinal oxalate absorption, e.g., inflammatory bowel disease, cystic fibrosis, biliary diseases, or chronic pancreatitis [61]. Additionally, hyperoxaluria has been associated with excessive vitamin C intake (metabolized in the liver to oxalates) and a diet rich in leafy vegetables (such as rhubarb and spinach), as well as coffee and cola drinks [62,63]. Interestingly, a recent study found that ingesting black tea does not increase the risk of stones [64].

To date, three distinct forms of primary hyperoxaluria have been identified: type I (#259900) related with biallelic pathogenic variants in the *AGXT* gene encoding alanine-glyoxylate aminotransferase and occurring in 70–80% of cases; type II (#260000) related with biallelic pathogenic variants in the glyoxylate reductase/hydroxypyruvate reductase *GRHPR* gene and responsible for 12% of PH cases, and type III (#613616) related to biallelic pathogenic variants in the HOGA gene, observed in 18% of PH cases [65]. Patients with PH1 (PH type I) develop severe nephrolithiasis and/or nephrocalcinosis, which typically results in irreversible renal damage and end-stage renal disease by the second decade of life. In end-stage renal disease, blood oxalate concentration increases, leading to oxalate-induced damage to the eyes, bones, and joints (oxalosis). Patients with PH2 and PH3 exhibit a milder phenotype, with the majority experiencing recurrent nephrolithiasis since childhood [65,66]. Until recently, the only curative treatment for PH1 was liver transplantation (LTx) or combined liver-kidney transplantation (CLKTx). However, both of these are associated with an increased mortality rate. The introduction of an innovative iRNA-based therapy, lumasiran, dramatically changed the natural course of PH1. Lumasiran was designed to silence the gene that encodes the enzyme glycolate oxidase, which catalyzes the conversion of glycolate into glyoxylate [67]. Lumasiran inhibits the progression of PH1. Data from both adults and children indicate the remarkable effectiveness of the drug in reducing oxalate concentrations in blood and urine, inhibiting the progression of nephrolithiasis and nephrocalcinosis, and, most importantly, maintaining kidney function [68,69,70]. Finally, lumasiran allowed for the performance of isolated kidney transplantation, instead of combined liver-kidney transplantation [71,72]. In Poland, treatment with lumasiran has been financed since 2022 by the Medical Fund as part of a dedicated drug program (National Health Fund therapeutic program) for patients with PH1 and chronic kidney disease of 1–3 degrees.

A 2019 study provides additional data on long-term outcomes in patients with PH2. The authors analyzed 101 patients with genetically confirmed PH2 from the OxalEurope registry. Twelve patients were lost to follow-up; 45 of the remaining 89 patients had CKD stage 2 or greater, and 22 patients had ESKD. Interestingly, there was no correlation between genotype, baseline biochemical parameters, or the presence of nephrocalcinosis and kidney outcome. The authors concluded that primary hyperoxaluria type 2 carries a significant renal morbidity. [73]. Similarly, analysis of 95 patients with PH3 from the same registry revealed that 21.6% had CKD stage 2 or greater. According to the researchers, PH3, despite being the most favorable PH type, is not a benign entity as it constitutes an early onset, recurrent stone disease, and may lead to kidney function impairment [74]. The outcome of enteric hyperoxaluria is highly dependent on intestinal disease, its treatment, and prognosis. A recently published study on kidney transplant recipients due to secondary hyperoxaluria showed that compared to reflux nephropathy, hyperoxaluria was associated with inferior graft survival [75].

As for diagnostics, recently published guidelines by ErkNET and OxalEurope recommend that, in all suspected children (i.e., all children with urolithiasis and all children with CKD of unknown origin), urinary oxalate excretion, along with creatinine, be assessed by 24 h urine collection. Spot urine collections may be used in place of 24 h urine collections where clinically necessary, with oxalate assessment expressed as the oxalate-to-creatinine ratio. The experts suggest that at least two positive urine assessments (urine oxalate higher than the upper reference limit) are required to establish hyperoxaluria, and that age-related reference values should be used (see Table 3). The experts also suggest measuring urinary metabolites (glycolate, L-glycerate, HOG, DHG) in patients with hyperoxaluria, as increased urinary glycolate suggests PH1, increased urinary L-glycerate—PH2, and increased urinary HOG and DHG—PH3 [67]. Experts highlight the need for genetic testing of each patient with high clinical and/or biochemical suspicion of PH, especially those with substantially increased urinary oxalate excretion [67,76]. The result of genetic testing carried out in a certified laboratory constitutes a definitive diagnosis of PH, as genetically negative cases are rare (provided that acquired forms of hyperoxaluria are excluded) [76].

### 5.3. Cystinuria

Cystinuria (#220100); excessive urine excretion of a dipeptide consisting of two cysteine molecules). It is one of the most prevalent genetic stone disorders, with an estimated prevalence of 1 in 7000 [77]. The 5-year recurrence rate for cystine stones is 83%, which is higher than noted for any other type of stone [29,78]. The disorder is a recessively inherited defect in a transporter located in the enterocytes and proximal tubules (though some patients may have an autosomal dominant pattern of inheritance). The disease is caused by pathogenic variants in the *SLC3A1* and *SLC7A9* genes, the heavy subunit (rBAT) and the light subunit (b0,+AT), respectively, of the heterodimeric transporter. A deficiency of the transporter in the intestine is not clinically relevant; however, its absence in the proximal tubule leads to the inhibition of reverse resorption of dibasic amino acids (cystine, lysine, arginine, and ornithine). Homozygotes experience severe, recurrent nephrolithiasis from an early childhood. The condition is exacerbated by urinary acidification and a diet high in meat and eggs, which contain sulfur-containing amino acids [79].

The diagnosis of cystinuria can be established by analysis of kidney stone composition, observation of cystine crystals in the urinary sediment, or detection of an elevated cystine urinary excretion. The demonstration of elevated cystine (and other dibasic amino acids: ornithine, lysine, and arginine) in a 24 h urine collection is sufficient to make a diagnosis of cystinuria. In small children, first- or second-morning urine samples can be used, and results should be reported as the cystine-to-creatinine ratio. The obtained result ought to be compared with pediatric reference values (Table 3). Monitoring urinary cystine is of limited value in the management of these patients. Of note, genetic tests are not mandatory. They are recommended in case of atypical presentation, uncertain mode of inheritance, and for genetic counseling and/or research purposes. The classic cyanide-nitroprusside urine test has poor reproducibility and sensitivity and is not recommended [79].

### 5.4. Hyperuricosuria

Hyperuricosuria (i.e., excessive excretion of uric acid in the urine) is observed in 2% to 8% of pediatric patients with nephrolithiasis [80]. However, pure uric acid stones in children are rare. The condition can result from a renal tubular defect (renal hyperuricosuria) or from systemic conditions that lead to excessive uric acid production, such as leukemia, lymphoma, gout, Lesch–Nyhan syndrome (#300322), cystic fibrosis, and glycogen storage disorders (GSD). Lesch–Nyhan syndrome is caused by a deficiency in hypoxanthine-guanine phosphoribosyltransferase (HPRT) activity and is associated with excessive uric acid production. Besides the presence of lithiasis and gout, various neurological abnormalities are observed, including severe action dystonia, choreoathetosis, ballismus, cognitive and attention deficit, and self-injurious behaviors. Among other inborn errors of metabolism related to hyperuricosuria, GSD types Ia and Ib (#232200) are characterized by decreased expression and overproduction of uric acid. The clinical manifestation of GSD type I includes the presence of hepatomegaly with increased transaminase activity (increased risk of developing liver adenoma, less frequently hepatocellular carcinoma) and severe hypoglycemia with concomitant lactic acidosis. The kidney damage progresses over time and manifests as both tubulopathy (damage to the renal tubules) and glomerulopathy (hyperfiltration, hypertension). Hypercalciuria and hypocitraturia are also observed in GSD type I, which increases the risk of nephrocalcinosis and urolithiasis. Patients with GSD Ib also have neutropenia and impaired neutrophil function, which predispose them to recurrent bacterial and fungal infections and oral ulcers.

Hyperuricosuria may also be observed in individuals consuming a diet high in purines, including meat broths, coffee, and tea. The crystallization of uric acid is exacerbated by low urinary pH and reduced urine volume, which can occur in warm climates or as a result of chronic diarrhea [81].

To assess uric acid excretion in urine, a 24 h urine collection should be performed or, in young children, uric acid excretion should be evaluated in a urine sample (in relation to creatinine) (Table 3). It is essential to simultaneously assess serum uric acid concentration to detect hyper- or hypouricemia. The calculation of fractional uric acid excretion is of great value—hypouricemia (<2 mg/dL) and increased fractional uric acid excretion (>11.1%) indicate renal hypouricemia [82]. As mentioned below, deposits composed of purine metabolites are radiolucent and difficult to visualize in X-ray-based examinations.

### 5.5. Xanthinuria and Other Promoters

Rare causes of nephrolithiasis include congenital defects in purine metabolism. In type I xanthinuria (XAN1; #278300), there is an isolated deficiency of xanthine dehydrogenase, and in type II xanthinuria (XAN2; #603592), there is a dual deficiency of xanthine dehydrogenase and aldehyde oxidase (due to lack of a common sulfido-molybdenum cofactor). Patients with xanthinuria typically present with hypouricemia, hypouricosuria, and elevated urinary xanthine excretion, with xanthine nephrolithiasis occurring in approximately one-third of affected individuals. In type II deficiency, some neurological manifestations have been described, including intellectual disability and autistic features. Urinary symptoms of xanthinuria are related to stone formation. Acute kidney injury or progression to chronic kidney disease is uncommon. The disease is often asymptomatic, and the detection of hypouricemia, orange-brown urinary sediment, or orange-stained diapers prompts diagnostic testing. Sometimes renal colic or obstructive nephropathy may be the first symptoms [83].

Adenine phosphoribosyl transferase (APRT) deficiency, also inherited in an autosomal recessive pattern, leads to the formation of deposits composed of 2,8-dihydroxyadenine. APRT deficiency is characterized by a highly variable clinical picture with no genotype-phenotype correlation. First of all, the age of first presentation can vary from infancy to adulthood. The disease may manifest as urolithiasis, orange- or reddish-brown-stained diapers, recurrent urinary tract infections, or acute kidney injury due to obstructive nephropathy. Interestingly, the disease may manifest as kidney failure in adulthood in the absence of visible stones due to tubular deposits of 2,8-DHA. Over 80% of patients with APRT deficiency, who have undergone kidney transplantation, develop crystal nephropathy. Thus, many are diagnosed after transplantation [4,84].

Additionally, there are case reports of nephrolithiasis due to the accumulation of pathological metabolites in patients with type I orotic aciduria and alkaptonuria [85].

Xanthinuria is initially suspected based on the presence of kidney stones and low concentrations of uric acid in the blood and urine. An allopurinol test may be helpful. If a patient is given allopurinol (a xanthine oxidase inhibitor) and oxypurinol is detected in their blood, it confirms Type 1 xanthinuria. As for diagnostic tests, normal values of serum xanthine and hypoxanthine are <9.0 µmol/L and <10 µmol/L, respectively, and normal urinary indices are: xanthine–creatinine ratio—<25 µmol/mmol and hypoxanthine–creatinine ratio—<30 µmol/mmol [86]. The analysis of the urine metabolome using gas chromatography–mass spectrometry (GC/MS) or ultra-high performance liquid chromatography–tandem mass spectrometry (UPLC-MS/MS) assays may detect adenine, 8-hydroxyadenine, and 2,8-dihydroxyadenine, which are undetectable in healthy and heterozygous controls. Substantially decreased or absent APRT activity in red blood cell lysates, or the detection of 2,8-dihydroxyadenine in plasma, can serve as another diagnostic tool. The diagnosis of these rare forms of urolithiasis can be confirmed by molecular testing [84]. Biallelic pathogenic variants in *XDH* (type I xanthinuria), *MOCOS* (XAN2), *APRT* (APRT deficiency), *UMPS* (type I orotic aciduria), and *HGD* (alkaptonuria) confirm the diagnosis.

### 5.6. Hypocitraturia

Hypocitraturia is a common co-occurring metabolic abnormality observed in pediatric and adolescent populations with nephrolithiasis. The function of citrates is, among other things, to form soluble complexes with calcium, thereby reducing urine supersaturation with calcium and inhibiting the nucleation and crystallization of calcium oxalate and calcium phosphate. Additionally, citrate acts as a buffer and alkalinizes the urine, increasing the solubility of weak organic acids such as cystine and uric acid. The prevalence of hypocitraturia has been reported to range from 10% to 64% among stone formers (mainly calcium-containing stones) [87], was statistically more frequent in adults than in children [87], and was statistically more frequent in adults than in children [87].

The substantial variability in incidence rates can be attributed to several factors, including the use of different reference ranges for urinary citrate across studies and differences in the type of urine samples analyzed, specifically random versus 24 h urine collections [55]. Hypocitraturia has been observed in several conditions characterized by chronic acidification, including the ketogenic diet, the use of carbonic anhydrase inhibitors such as acetazolamide, and a range of tubular acidoses, particularly distal tubular acidosis [88]. Moreover, a correlation has been established between a reduction in dietary intake of potassium and magnesium and the occurrence of hypocitraturia [89]. Hypokalemia causes both intracellular acidosis and a decrease in tubular pH, resulting in increased citrate uptake and metabolism [88]. Conversely, the administration of magnesium salts has been shown to increase urinary pH and citrate excretion [90]. The sodium-dependent dicarboxylate transporter 1 (NaDC-1) is the most significant protein responsible for citrate reabsorption. This protein is expressed in the proximal tubule, small and large intestine, liver, and brain. Calcium and magnesium bind to citrate in the urine, thereby reducing the pool of citrate available to the NaDC-1 transporter [91].

As already mentioned, hypocitraturia is associated with increased risk for calcium-oxalate and uric acid stone disease. A retrospective analysis revealed a significant increase in hypocitraturia prevalence among stone formers since the 1980s, from 46% to 60%. This trend parallels the increased prevalence of obesity, diabetes mellitus, and metabolic syndrome [92].

To assess citrate excretion in urine, as with other molecules, a 24 h urine collection is used; in small children or children with urinary incontinence, the citrate-to-creatinine ratio is used. There are doubts regarding the reference values for urinary citrate excretion in children. Hoppe gives higher cut-off values for boys and girls than the Polish standards (which Prof. Hoppe also co-authored) [29,53,55]. Table 3 shows the most commonly accepted reference values. In patients with hypocitraturia, it is essential to assess blood and urine pH to check for systemic acidosis (both blood and urine will be acidotic) and complete and incomplete distal renal tubular acidosis (blood pH will be acidotic or normal, and urine pH will be > 5.5).

## 6. Other Factors Predisposing to Urolithiasis

### 6.1. Infectious Urolithiasis

Infectious urolithiasis refers to a form of urinary stone disease in which chronic bacterial infection or colonization of the urinary tract plays a significant role in its pathogenesis. Bacteria that possess the enzyme urease—such as *Proteus* spp., *Klebsiella* spp., *Providencia* spp., *Pseudomonas* spp., *Morganella morganii*, and *Staphylococcus aureus*—hydrolyze urea into ammonia and carbon dioxide. This reaction leads to the formation of ammonium ions and the alkalinization of the urine, a process that facilitates the precipitation of ammonium magnesium phosphate (struvite), carbonate apatite, or hydroxyapatite. These infectious stones tend to grow rapidly, often filling the entire renal pelvis (commonly referred to as staghorn calculi), and are associated with a high risk of recurrence [93,94].

In children, this disease is observed in 1% to 29% of patients with urolithiasis. As in adults, some studies indicate a higher incidence of struvite urolithiasis in girls [95]. It is also more common in younger children, which seems to be related to the more frequent occurrence of infections and urinary tract defects in this group of patients [96]. In the pediatric population, struvite urolithiasis occurs most commonly in patients with other predisposing factors, such as a neurogenic bladder or urinary tract malformations (vesicoureteral reflux, posterior urethral valves, ureterovesical junction obstruction) [93,97]. Urinary tract defects are found in 1 in 3 children with struvite urolithiasis [6]. Struvite and apatite urolithiasis of the urinary bladder are the most common forms of stones in children with neurogenic bladder who undergo clean intermittent catheterization (CIC)—including children after surgical bladder augmentation [98]—as well as in patients with a urinary reservoir created from the intestine (e.g., Bricker pouch). In all these cases, chronic or recurrent bacterial urinary tract infection is considered the causative factor in urolithiasis.

The symptoms of infectious urolithiasis combine those of urolithiasis and urinary tract infection (fever, dysuria, lumbar pain, urinary urgency). Typical symptoms of renal colic are rare [99]. Urinalysis reveals an alkaline reaction, the presence of nitrites and a positive reaction to leukocyte esterase, and, in the sediment, crystals of ammonium magnesium phosphate, in addition to signs of infection (leukocyturia, sometimes leukocyte casts). These crystals most often take the shape of a coffin lid; less frequently, a fern leaf. Urine culture is positive for urease-producing bacteria. Imaging studies usually reveal a large branched calculus. If appropriate treatment is not implemented, it fills the entire renal pelvis and calyces [93,99].

Chronic infectious urolithiasis leads to recurrent pyelonephritis, with scarring of the renal parenchyma and progression to CKD and even ESKD. Data collected from adult patients indicate that struvite urolithiasis is the most common type of urolithiasis to lead to kidney failure [100]. In addition, bacterial infection can result in sepsis. Spontaneous expulsion of struvite stones is extremely rare. The prognosis in this group of patients depends entirely on the complete removal of the stone and no recurrence. The percentage of patients free of recurrence and infection reaches 90% if the stones are completely removed during the first procedure [93]. If a fragment of the stone is left behind, the risk of recurrence of clinically apparent urolithiasis ranges from 40% to 85% [93,99].

### 6.2. Urinary Structural Abnormalities

Congenital anatomical abnormalities of the urinary system are a significant predisposing factor for urolithiasis, observed in 11–30% of pediatric patients with the condition [101,102]. However, only 1% to 5% of children with such urologic abnormalities go on to develop calculi, which suggests the presence of an additional metabolic abnormality in patients who exhibit both urologic anomalies and stone formation. Particularly significant abnormalities associated with urinary stasis include vesicoureteral reflux, ureteropelvic junction obstruction in 3 ureterovesical junctions, and neurogenic bladder [26,103]. Especially in obstructive abnormalities, urinary flow is impeded, predisposing the patient to increased solute precipitation in the urine.

Neurogenic bladder (especially as a consequence of spina bifida) carries the highest risk for urolithiasis—up to 36% percent (adult data) [104]. Analysis of 111 children with urolithiasis from France revealed that congenital anomalies of the kidney and/or urinary tract (CAKUT) or neurogenic bladder were present in 9/24 (38%) patients with infectious stones and 12/16 (76%) patients with bladder stones. [102]. Sometimes urolithiasis may be the first symptom of CAKUT or neurogenic bladder. If abnormalities are found in imaging tests performed for urolithiasis (ultrasound, computed tomography), further diagnostic tests are necessary—voiding cystourethrography, renal scintigraphy, uro-tomography, or uro-resonance may be required.

### 6.3. Acquired Risk Factors

Among the acquired risk factors for urolithiasis, the following should be highlighted: obesity (leading to increased production of uric acid and reduced urinary inhibitors), a diet rich in fructose (fructose metabolism consumes large amounts of adenosine triphosphate—ATP, which releases uric acid), ketogenic diet, a diet high in vitamins C and D, salt, low fluid intake, the use of corticosteroids and loop diuretics (which increase urinary calcium excretion), topiramate and acetazolamide (inducing metabolic acidosis and thereby reducing urinary citrate levels), acyclovir and ceftriaxone (due to drug crystallization in the urinary tract), and immobilization [105,106]. The summary and mechanisms by which environmental factors increase the risk of urolithiasis are presented in Table 5.

Adult epidemiological data indicate that increased body weight and higher body mass index are associated with higher urinary concentrations of calcium, oxalate, and uric acid and lower urinary pH [16]. Similarly, in children, obesity, metabolic syndrome, arterial hypertension, and metabolic syndrome were all found to be associated with increased risk for supersaturation of urine and urolithiasis [17,107]. Surprisingly, some pediatric data do not confirm the relationship between BMI and risk for urolithiasis in children [108].

The ketogenic diet, which is recognized as an effective treatment for drug-resistant epilepsy, is associated with numerous systemic risk factors: acidification of the body and urine, reduced citrate concentration in urine (resulting in acidification, among other things), increased excretion of uric acid in urine and an increased risk of its crystallization, as well as bone resorption and increased calcium concentration in urine. All these abnormalities pose a very significant risk of kidney stones, which is why many scientific societies recommend citrate supplementation from the moment a ketogenic diet is implemented. In a US study published in 2000, 6 out of 112 children treated with a ketogenic diet developed kidney stones, with hypercalciuria and younger age being risk factors [105]. In contrast, in a Turkish study published in 2023, 15 out of 95 children treated with this diet developed kidney stones. Interestingly, in this cohort, age, gender, calciuria, and the use of additional anti-epileptic drugs did not affect the risk of stone formation [109]. It should be noted that carbonic anhydrase inhibitors (e.g., acetazolamide) work similarly. Furthermore, modifications to the ketogenic diet (rich in protein, with extreme carbohydrate restriction) are now commonly used among teenagers who wish to lose weight.

## 7. Nephrocalcinosis

Nephrocalcinosis refers to the deposition of calcium salts within renal tissue. With the increasing frequency of ultrasonographic examinations in children, nephrocalcinosis is often detected incidentally. It can be classified into medullary and cortical types. Medullary nephrocalcinosis is associated with conditions such as congenital tubulopathies that increase tubular calcium secretion, for example, distal renal tubular acidosis (dRTA) or familial hypomagnesemia with hypercalciuria and nephrocalcinosis (FHHNC). Cortical nephrocalcinosis may result from events such as renal vascular thrombosis. Risk factors for nephrocalcinosis also include prematurity and vitamin D3 overdose [110,111].

In some patients, nephrocalcinosis may coexist with nephrolithiasis—deposits in such patients accumulate in the renal parenchyma and renal tract. This situation is associated with the risk of progressive kidney damage. It is observed, among others, in primary hyperoxaluria type I or certain forms of hypercalciuria (e.g., in the course of pathogenic variants of *SLC34A1* or *SLC34A3*) [67,112].

Of note, many genetically inherited diseases leading to nephrocalcinosis may have an unfavorable outcome and can progress to kidney failure with arterial hypertension, proteinuria, and even ESKD. As these are all rare entities, this poor outcome was unmasked in multicenter observational studies, for example, FHHNC (due to *CLDN16* biallelic pathogenic variants) [104], *SLC34A1*- and *SLC34A3*-related nephrocalcinosis [103], Lowe syndrome, and Dent disease [113].

Each case of nephrocalcinosis requires a thorough diagnostic evaluation in an experienced nephrology unit with access to molecular diagnostics.

**Figure 1:**
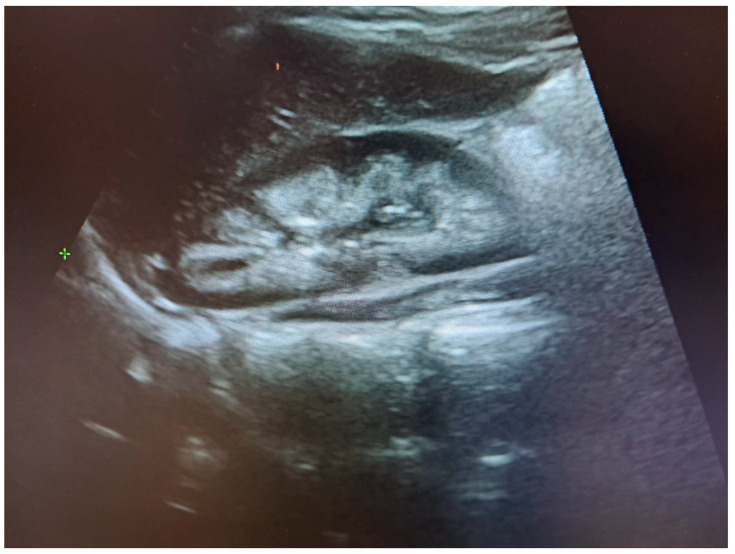
Nephrocalcinosis on ultrasonography.

The image of nephrocalcinosis on the ultrasonographic examination is displayed in Figure 1.

## 8. Diagnosis

**Figure 2:**
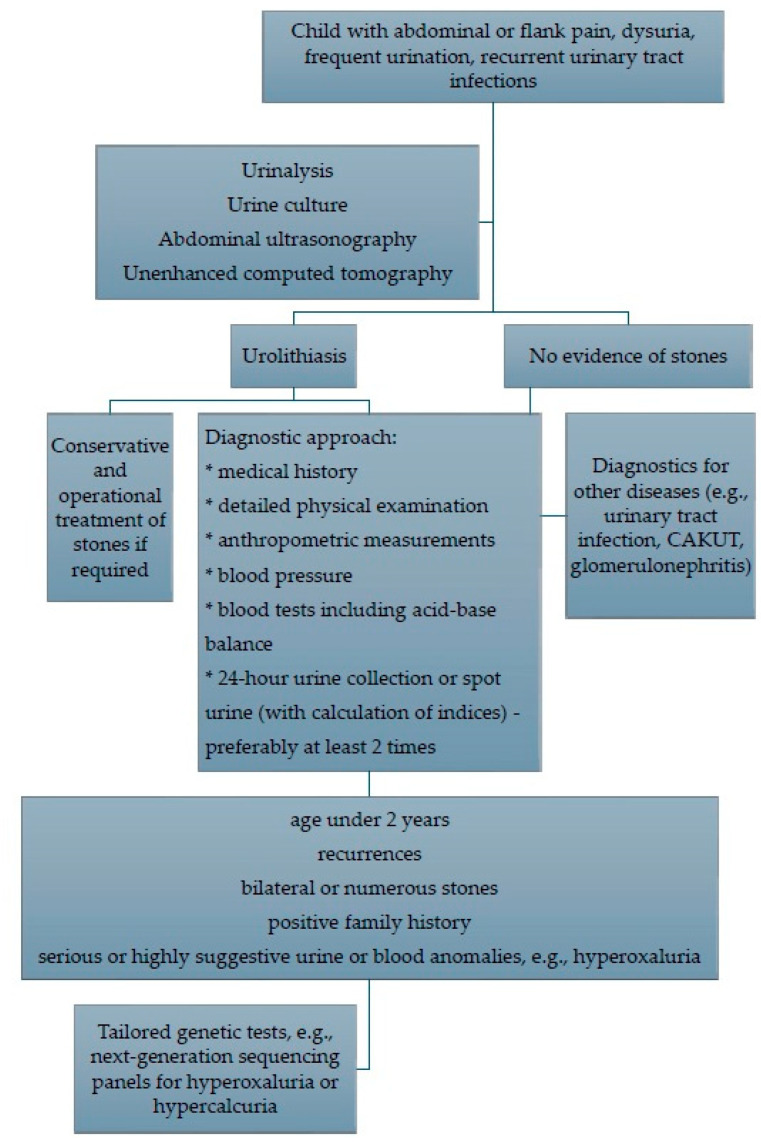
Proposed diagnostic approach to a child with urolithiasis.

Proposed diagnostic steps for a child with urolithiasis are presented in Figure 2.

### 8.1. History and Physical Examination

We distinguish between an acute renal colic attack, a state of increased risk for urolithiasis, and abnormalities detected in urine analysis or ultrasound examination [28]. The symptoms of urolithiasis in children are presented in Table 6.

**Table 6:** Symptoms of urolithiasis in children [7,26,28,36].

Clinical Symptoms of Urolithiasis
Abdominal, flank, or pelvis pain
Acute pain radiating to the groin, testicles, and labia majora
Dysuria
Frequent micturition
Polyuria
Enuresis
Nycturia
Macroscopic hematuria
Urgency
Nausea
Vomiting
Hypotension and fainting induced by pain
Loss of appetite
Anxiety
Diarrhea

It is worth noting that typical renal colic only occurs in older children. The youngest children may present with very non-specific symptoms such as irritability or tearfulness. Young children may indicate abdominal pain that is not necessarily located in the lumbar region. Up to 40% of infants may be asymptomatic, and urolithiasis is detected during diagnostic tests (most often abdominal ultrasound) performed for other reasons. Recurrent urinary tract infections may be an essential symptom in the youngest children and may prompt the initiation of urinary tract diagnostics [114].

As with any other disease, the diagnostic process begins with taking a thorough medical history. It is essential to inquire about symptoms indicative of urolithiasis (e.g., abdominal pain, signs of renal colic, urinary tract infections), other kidney diseases (including urinary tract malformations), and chronic diarrhea (which may suggest undiagnosed inflammatory bowel disease and episodes of dehydration). Additionally, the presence of pathological fractures should be noted, as they may suggest generalized calcium-phosphate metabolism disorders, such as primary hyperparathyroidism. A detailed analysis of the patient’s diet, fluid intake (type and quantity), and medications, including vitamin supplements (notably vitamins C and D), is essential. A comprehensive family history is also significant. The physical examination should be thorough, with special attention to the genitourinary system (as anatomical abnormalities may suggest underlying urinary tract malformations). Additionally, signs of growth retardation and rickets (which may indicate tubulopathies such as distal renal tubular acidosis) should be carefully evaluated. Abdominal examination and assessment for the presence of the Goldflam sign (tenderness upon percussion of the renal area, which suggests nephrolithiasis with impaired urine outflow or renal parenchymal infection) are also crucial components of the examination [8,26,36].

When examining a patient with urolithiasis, one should not overlook the ‘red flags’ that require immediate treatment, even before a preliminary metabolic diagnosis. Such ‘red flags’ include fever, especially when laboratory findings suggest active infection (leukocyturia, nitrites, positive urine culture) and a suspected or confirmed obstructing stone. In such cases, rapid conservative and surgical urological treatment is necessary. Clinicians may encounter this situation in any pediatric urolithiasis, especially in infectious urolithiasis.

### 8.2. Diagnostic Imaging

**Figure 3:**
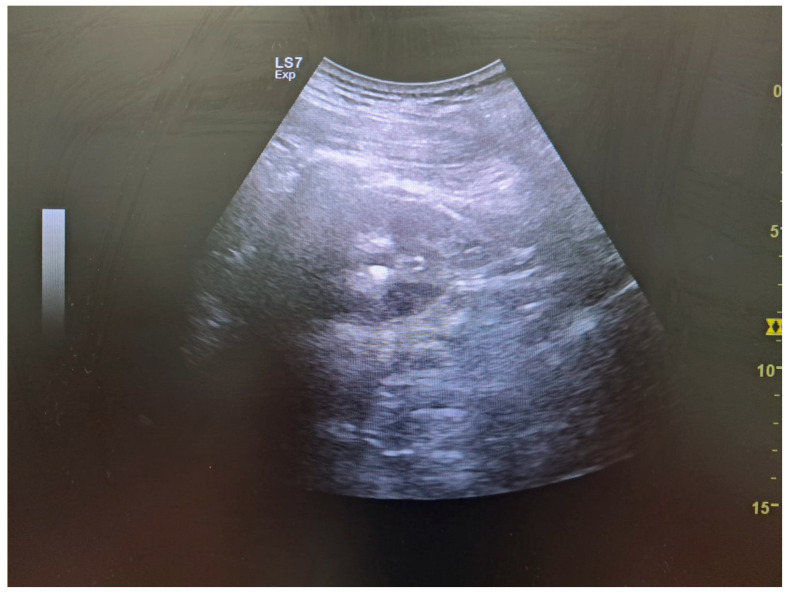
Kidney stone and acoustic shadow behind it (ultrasonography).

In contrast to adults, where non-contrast CT is the preferred method for diagnosing urolithiasis, ultrasound (US) is recommended as the initial diagnostic tool in children due to its non-ionizing nature [115]. The advantages of ultrasound include its repeatability, independence of renal function, and the absence of special preparation. Although ultrasound accuracy, in terms of sensitivity and specificity, can be influenced by the operator’s skill, equipment quality, and patient positioning. In ultrasound imaging, urinary tract calculi can be visualized. In doubtful cases, indirect assessment should be employed (such as evaluation of the acoustic shadow behind the calculus (Figure 3) or twinkling artifact). It should be emphasized that the middle part of the ureter is usually not visualized in ultrasonography. Therefore, in the presence of a stone in this part of the urinary tract, only dilation of the upper urinary tract may be observed [116,117,118].

In cases where a calculus is not visible despite a high likelihood of urolithiasis, it is advised to proceed with computed tomography (CT) without contrast as a secondary diagnostic tool. In radiographic (X-ray) or CT imaging, so-called radiopaque calculi, which contain metal cations (such as calcium oxalate, calcium phosphate, struvite, or hydroxyapatite), are easily visualized (Table 7). In contrast, calculi composed of different substances (such as uric acid, xanthine, 2,8-dihydroxyadenine, or cystine) are less readily detected [116,117].

**Table 7:** Radiopaque and radiolucent calculi [116].

Radiopaque Calculi	Radiolucent Calculi
calcium oxalate calcium phosphate struvite hydroxyapatite	uric acid xanthine 2,8-dihydroxyadenine cystine

Magnetic resonance imaging (MRI) currently plays a limited role in diagnosing urolithiasis, despite its advantages as a non-invasive, radiation-free imaging technique. MRI is highly sensitive and provides comprehensive anatomical detail through three-dimensional (3D) visualization, which aids in the detection of urinary tract malformations. Additionally, MR urography allows assessment of renal function by analyzing parenchymal uptake of the contrast agent and urinary excretion. Traditional intravenous urography has also mainly been replaced by CT scanning in this diagnostic context [116,117,118].

In summary, it should be noted that, in accordance with the ALARA (as low as reasonably achievable) principle and the recommendations of the European Association of Urology, ultrasound examination is the method of choice in children. However, a significant limitation of this technique is its inability to visualize mid-ureter stones. Therefore, in all doubtful cases, radiographic techniques are preferred, with unenhanced CT on low-dose protocols as the primary modality [119,120].

### 8.3. Blood Tests

In all patients with urolithiasis, a series of blood tests should be performed, including a complete blood count, a full electrolyte panel (sodium, potassium, calcium, phosphorus, magnesium, chloride), serum levels of creatinine, urea, and uric acid, and capillary blood gas analysis. The presence of hypokalemia, hyperchloremia, and metabolic acidosis is indicative of renal tubular acidosis. Given that the majority of cases involve calcium-based calculi, a comprehensive evaluation of calcium-phosphate metabolism is essential, including measurements of calcium, phosphorus, 25-hydroxyvitamin D, parathyroid hormone, and alkaline phosphatase activity (see Section 5.1).

Furthermore, measurement of 1,25(OH)_2_D is of diagnostic value, as elevated levels are observed in HCINF1 and in nephrolithiasis associated with increased 1-alpha-hydroxylase activity. These conditions include, as already mentioned, patients with pathogenic variants in SLC34A1 and SLC34A3 (enzyme uncoupling caused by low FGF23 concentrations) and patients with granulomatous diseases (macrophages expressing this enzyme), such as sarcoidosis [121]. As diagnosing these conditions is essential, measuring 1,25(OH)_2_D is of great practical significance. On the other hand, the limitations of this test should be kept in mind: 1,25(OH)_2_D concentration is not a good marker of vitamin D supply to the body, has a short circulating half-life, and its concentration reflects only recent physiological activity rather than long-term status. And finally, the molecule’s lipophilic nature and low serum concentration still pose analytical challenges and require complex sample preparation or highly sensitive equipment [122].

### 8.4. Urine Analysis

In the general urinalysis of a child with urolithiasis, the most commonly observed abnormality is hematuria. Additionally, reactive proteinuria and leukocyturia (resulting from urinary tract irritation (sterile) or as a sign of urinary tract infection) may be present. Depending on the pH, different chemical compounds crystallize [123]. However, the presence of crystals in the urine is not diagnostic of urolithiasis, except when hexagonal cystine crystals are present. The identification of such crystals is an unequivocal indication of cystinuria. Confirmation of cystinuria is achieved by detecting elevated cystine concentrations in a spot urine or 24 h urine collection. In a child with suspected urolithiasis, a urine culture should be performed to rule out a urinary tract infection. The presence of urease-producing bacteria in the urine is indicative of infection-related urolithiasis [124,125].

### 8.5. Evaluation of Stone Composition

To prevent the recurrence of urolithiasis, it is essential to identify the type of stone present. The most valuable diagnostic method in this regard is the analysis of the stone’s composition excreted in the urine. In instances of initial urolithiasis, it is crucial to ascertain the stone’s composition. It is therefore recommended that patients be instructed to strain their urine using gauze. For young children who have not yet developed bladder control, caregivers should inspect diapers closely. The accepted methods for stone composition analysis include infrared spectroscopy (IRS), X-ray diffraction (XRD), polarizing microscopy, and chemical analysis. Of these, infrared spectroscopy is a continually advancing technique and is considered the gold standard in stone composition analysis [126]. Both infrared spectroscopy and X-ray diffraction are recommended by the European Association of Urology (EAU) and a consensus conference in 2021 [79,120,124,127]. In contrast, chemical analysis is currently regarded as outdated, leading to the destruction of the calculus and preventing analysis by other methods [124,128].

### 8.6. The 24 h Urine Analysis

The consensus group of nephrologists, urologists, and clinical biologists highlighted that 24 h urine collection is the most effective method for urine collection for testing [124]. In a 24 h urine collection, we assess the concentrations of creatinine, calcium, phosphates, uric acid, magnesium, sodium, as well as citrate and oxalate levels. In adult patients, a reduction in calcium oxalate (CaOx) supersaturation, along with decreased 24 h urinary excretion of citrate, potassium, and magnesium during treatment, correlated with extended recurrence-free intervals [129]. To accurately evaluate these parameters, it is essential to conduct testing under specific conditions. These include excluding any urinary tract infection and removing any calculi from the urinary system [124]. The American Urological Association suggests performing two 24 h urine collections. At the same time, the European Association of Urology recommends conducting a follow-up 24 h urine measurement eight to twelve weeks after initiating pharmacological prevention of stone recurrence [120,130]. It is also recommended that urine collection for analysis should occur at home, with the patient maintaining their typical diet and usual lifestyle. This approach minimizes the potential for measurement errors that may arise from transitioning to a hospital diet. Before analyzing the excretion of components in a 24 h urine collection, it is necessary to assess the completeness of the collection by calculating the total creatinine excretion. A result that falls outside the normal range (15–25 mg/kg/24 h) indicates that the 24 h urine collection may have been performed inaccurately [124].

In younger, mentally disabled, or diapered children, collecting all urine samples over a 24 h period can be challenging; therefore, estimations may be made using a single sample collected at a specified time of day. A recent study has shown that spot urine samples are equivalent to urine osmolality from 24 h urine samples [80], although recent normative values for the spot calcium– and phosphate–creatinine ratio were obtained from the second morning urine sample [54]. Additionally, in pediatric patients with urolithiasis, it is advisable to measure urine pH from the morning sample, or alternatively, to assess urine pH under paraffin or via a 24 h urine pH profile. A persistent urine pH above 5.5, particularly in conjunction with hypercalciuria and hypocitraturia, suggests incomplete distal renal tubular acidosis and warrants an acidification test using furosemide or ammonium chloride [131].

Regarding the evaluation of phosphate urine excretion, the recommendations emphasize the usefulness of indicators calculated from urine samples rather than daily urine collections [54]. To assess phosphate excretion in urine, calculation of the fractional excretion of phosphate (FePO_4_), tubular reabsorption of phosphate (TRP), and the TMP/GFR ratio are recommended. To calculate these indicators (together with the urinary calcium–creatinine ratio), the calculator available on the website of the European Society for Paediatric Nephrology (https://www.espn-online.org/tmp-gfr-calculator/#calculator) can be used. The authors used as reference values the already quoted standards of Pott et al. [54].

### 8.7. Genetic Testing

In many cases, once a specific metabolic defect is identified, the definitive diagnosis is established based on targeted genetic (molecular) testing. Such testing should be considered in children when there is clinical suspicion of an inherited metabolic disorder, including early age at onset of the disease (<2 years), recurrent disease (≥2 episodes), bilateral disease, or a concerning family history. In recent years, genetic testing using next-generation sequencing (NGS) technology has become increasingly accessible, enabling the detection of known monogenic causes of kidney stones and the identification of novel gene variants [132,133]. Monogenic causes of nephrolithiasis are listed in Table 8.

Cascade genetic testing, or testing of at-risk relatives, is extremely promising, as it offers genetic testing and potentially life-saving risk-reduction strategies to a population that is exponentially enriched for the risk of carrying a pathogenic variant. Cascade testing involves testing close family members, such as siblings, for a specific variant, and has already proven effective in hereditary cancer syndromes and cardiovascular diseases [134,135]. In nephrology, cascade testing is recommended, for example, in Alport syndrome [136]. Its usefulness has been demonstrated in families of children with kidney failure [137]. It seems that this method may also apply to the detection of monogenic kidney stone disease, enabling early implementation of preventive measures and preventing long-term adverse kidney sequelae.

## 9. Conclusions

Pediatric urolithiasis is often a manifestation of underlying renal or systemic abnormalities. Metabolic disturbances, particularly idiopathic hypercalciuria and hypocitraturia, are the leading contributors. Evaluation of a 24 h urine collection remains the gold standard for unmasking the underlying metabolic anomaly. Genetic testing is recommended in selected cases: disease starting in the first two years of life, recurrent, bilateral, or massive urolithiasis, positive family history, or severe anomalies detected in urine or blood tests. Identifying and addressing the root causes is critical to preventing recurrence and ensuring positive long-term health outcomes in pediatric patients. Given the high risk of recurrence and potential unfavorable renal outcome, long-term follow-up of all pediatric patients with urolithiasis is necessary.

## Figures and Tables

**Table 1:** Inhibiting and promoting factors of stone formation [30,33].

Inhibiting Factors	Promoting Factors
Citrate Magnesium Pyrophosphate Uromodullin (Tamm-Horsfall protein) Fetuin-A Nephrocalcin Osteopontin Lithostatine Bacunin Fibronectin Chondroitin sulphate Heparin sulphate Inter-alpha-inhibitor Urinary prothrombin fragment-1 Glycosaminoglycans	Calcium Oxalate Sodium Urate Cystine Nucleolin Myeloperoxidase

**Table 2:** The reference values for urinary calcium excretion.

Daily Urinary Collection	Urinary Calcium–Creatinine Ratio by Hoppe [53]		Urinary Calcium–Creatinine Ratio by Pott [54]	
Children: <4 mg/kg/24 h Adult females: >250 mg/24 h Adult males >300 mg/24 h	<1 y.o. 1–3 y.o. 3–5 y.o. 5–7 y.o. 7–17 y.o.	<0.81 <0.53 <0.39 <0.28 <0.21	0 y.o. 0.5 y.o. 1 y.o. 2 y.o. 5 y.o. 8 y.o. 10 y.o. 15 y.o. 18 y.o.	0.53 0.50 0.48 0.44 0.33 0.27 0.24 0.19 0.17

**Table 3:** Reference values for urinary excretion of mineral indicators according to [29,53,55,56] with own modifications. The reference values of calciuria by Pott are listed in Table 2.

Urine Component	24 h Urine Collection	Indicator from a Urine Sample (Normalized to Creatinine) (mg/mg Creatinine)	
Calcium	<4 mg/kg/24 h	<1 y.o. 1–3 y.o. 3–5 y.o. 5–7 y.o. 7–17 y.o.	<0.81 <0.53 <0.39 <0.28 <0.21
Magnesium	>2 y.o.—>88 mg/1.73 m^2^/24 h	0–1 y.o. 1–2 y.o. 2–3 y.o. 3–5 y.o. 5–7 y.o. 7–10 y.o. 10–14 y.o. 14–17 y.o.	>0.48 >0.37 >0.34 >0.29 >0.21 >0.18 >0.15 >0.13
Uric acid	<815 mg/1.73 m^2^/24 h or: <1 y.o.—<12.9 mg/kg/24 h 1–5 y.o.—<11 mg/kg/24 h >5 y.o.—<9 mg/kg/24 h	<1 y.o. 1–3 y.o. 3–5 y.o. 5–10 y.o. >10 y.o.	<2.2 <1.9 <1.5 <0.9 <0.6
Oxalate	<45 mg/1.73 m2/24 h (or: <0.5 mmol/1.73 m^2^/24 h)	<6 m.o. 6 mo.–2 y.o. 2–5 y.o. 6–12 y.o. >12 y.o.	<0.180 <0.140 <0.080 <0.063 <0.031
Citrates	Boys: >175 mg/1.73 m^2^ Girls: >253 mg/1.73 m^2^	0–5 y.o. >5 y.o.	>0.42 >0.25
Cystine	<10 y.o.—<13 mg/1.73 m^2^/24 h >10 y.o.—<48 mg/24 h	<1 y.o. 1–3 y.o. 3–5 y.o. 5–7 y.o. 7–17 y.o.	<0.81 <0.53 <0.39 <0.28 <0.21

Before analyzing the excretion of components in a 24 h urine collection, the completeness of the collection should be assessed by calculating creatinine excretion. A value outside the reference range (15–25 mg/kg/24 h) suggests an improperly conducted 24 h urine collection.

**Table 4:** Calcium–phosphorus metabolism in different causes of hypercalciuria [58,59,60].

Etiology	Ca	P	Mg	K	ALP	PTH	25OHD	1,25(OH)_2_D	U_Ca_	U_P_	TmP/GFR	U_Mg_
Primary hyperparathyroidism	↑	↓	N	N	↑	↑	N	N/↑	↑	↑	↓	N
Pathogenic variants in *SLC34A1* or *SLC34A3*	N/↑	↓/N	N	N	↑	N/↑	N	↑/N	↑/N	↑	↓	N
Pathogenic variants in *CYP24A1*	↑	↑	N	N	N	↓	↑	N/↑	↑	N/↑	N	N
Vitamin D overdosage	↑	↑	N	N	N	↓	↑	N/↓	↑	N/↑	N	N
Bartter syndrome	N/↓	N	N/↓	↓	N	N	N	N	↑	N	N	N/↑
Dent-1 and Dent-2 disease	N	N/↓	N/↓	N/↓	↑	N/↑	N	N/↑	↑	N/↑	N/↓	N/↑
Familial hypomagnesemia with hypercalciuria and nephrocalcinosis	N	N	↓	N	N	N/↑	N	N	↑	N	N	↑
Fanconi syndrome	N/↓	↓	↓	↓	↑	N/↑	N	N/↓	↑	↑	↓	↑

Ca—serum calcium, P—serum phosphate, Mg—serum magnesium, K—serum potassium, ALP—alkaline phosphatase, PTH—parathyroid hormone, 25OHD—25-hydroxyvitamin D, 1,25(OH)_2_D—1,25-dihydroxyvitamin (calcitriol), iFGF23—intact fibroblast growth factor 23, U_Ca_—urinary calcium, U_P_—urinary phosphate, TmP/GFR—tubular maximal phosphate reabsorption related to glomerular filtration rate, N—normal, ↓—lowered, ↑—elevate.

**Table 5:** Environmental factors predisposing to urolithiasis and the mechanisms by which they work.

Factor/Factors	Mechanism
Overweight/obesity, metabolic syndrome	Hypercalciuria Hyperuricosuria Hyperoxaluria Hypocitraturia Acidifying urine pH
High-salt (sodium) diet	Hypercalciuria
Low fluid intake	Low urine output and supersaturation
Hot climate	Low urine output and supersaturation
Immobilization	Bone resorption Hypercalciuria
Ketogenic diet and its modification	Hypercalciuria Hyperuricosuria Hypocitraturia Metabolic acidosis Acidifying urine pH
Carbonic anhydrase inhibitors	Hypercalciuria Hypocitraturia Metabolic acidosis
Vitamin C supplementation	Hyperoxaluria
Vitamin D supplementation	Hypercalciuria
Sulfonamides, ceftriaxone, acyclovir, indinavir, atazanavir, nelfinavir, triamterene, methotrexate	Crystallization in the urinary tract
Loop diuretics, e.g., furosemide	Hypercalciuria
Corticosteroids	Hypercalciuria

**Table 8:** Monogenic causes of urolithiasis, according to [29], with our own modifications.

Predisposing Conditions for Nephrolithiasis	Genetic Defects
Hypercalciuria	• Dent Disease type 1 (*CLCN5*)—XR • Dent Disease type 2/Lowe Syndrome (*OCLR*)—XR • Autosomal Dominant Hypocalcemia with Hypercalciuria (*CASR*)—AD • Familial Hypomagnesemia with Hypercalciuria and Nephrocalcinosis • (*CLDN16*, *CLDN19*)—AR • Infantile Hypercalcemia type 1 (*CYP24A1*)—AR * • Infantile Hypercalcemia type 2 (*CYP34A1*)—AR * • Bartter Syndrome type 1–5 (*SLC12A1*, *KCNJ1*, *CLCNKB*, *BSND*, *MAGED2*)—AR, XR (type 5) • Familial Hypophosphatemic Rickets with Hypercalciuria (SLC34A3)—AR * • Pseudohypoaldosteronism type 2 (*WNK4*)—AD • Transaldolase deficiency (*TALDO1*)—AR
Hyperoxaluria	• Primary Hyperoxaluria type 1 (*AGXT*)—AR • Primary Hyperoxaluria type 2 (*GRHPR*)—AR • Primary Hyperoxaluria type 3 (*HOGA1*)—AR
Hypocitraturia	• Distal Renal Tubular Acidosis (*SLC4A1*)—AD/AR, (*ATP6V1B1*)—AR, (*ATP6V0A4*)—AR
Hyperuricosuria and Other Purine Metabolism Defects	• 2,8-Dihydroxyadenine Stone Formation (*APRT*)—AR • Type I xanthinuria (*XDH*)—AR • Type II xanthinuria (*MOCOS*)—AR • Renal Hypouricemia (*SLC22A12*)—AR • Lesch–Nyhan Syndrome (*HPRT1*)—XR • Glycogen Storage Disease type 1a (*G6PC*)—AR • Glycogen Storage Disease type 1b (*SLC37A4*)—AR
Cystinuria	• Cystinuria type 1 (*SLC3A1*)—AR • Cystinuria type 2 (*SLC7A9*)—AR
Other	• Type I orotic aciduria (*UMPS*)—AR • Alkaptonuria (*HGD*)—AR

AR—Autosomal Recessive Inheritance, AD—Autosomal Dominant Inheritance, XR—X-linked Recessive Inheritance. * Hypercalciuria may also occur in heterozygotes.

## Data Availability

No new data wealyzed in this study. Data sharing is not applicable to this article.
